# The THO/TREX Complex Component RAE2/TEX1 Is Involved in the Regulation of Aluminum Resistance and Low Phosphate Response in *Arabidopsis*

**DOI:** 10.3389/fpls.2021.698443

**Published:** 2021-07-12

**Authors:** Yi-Fang Zhu, Jinliang Guo, Yang Zhang, Chao-Feng Huang

**Affiliations:** ^1^College of Resources and Environmental Sciences, Nanjing Agricultural University, Nanjing, China; ^2^Shanghai Center for Plant Stress Biology and National Key Laboratory of Plant Molecular Genetics, CAS Center for Excellence in Molecular Plant Sciences, Chinese Academy of Sciences, Shanghai, China

**Keywords:** aluminum resistance, *Arabidopsis thaliana*, low phosphate response, STOP1, TEX1, THO/TREX complex

## Abstract

The C2H2-type zinc finger transcription factor SENSITIVE TO PROTON RHIZOTOXICITY 1 (STOP1) plays a critical role in aluminum (Al) resistance and low phosphate (Pi) response mainly through promoting the expression of the malate transporter-encoding gene *ARABIDOPSIS THALIANA ALUMINUM ACTIVATED MALATE TRANSPORTER 1 (AtALMT1)*. We previously showed that REGULATION OF ATALMT1 EXPRESSION 3 (RAE3/HPR1), a core component of the THO/TREX complex, is involved in the regulation of nucleocytoplasmic *STOP1* mRNA export to modulate Al resistance and low Pi response. Here, we report that RAE2/TEX1, another core component of the THO complex, is also involved in the regulation of Al resistance and low Pi response. Mutation of *RAE2* reduced the expression of STOP1-downstream genes, including *AtALMT1*. *rae2* was less sensitive to Al than *rae3*, which was consistent with less amount of malate secreted from *rae3* roots than from *rae2* roots. Nevertheless, low Pi response was impaired more in *rae2* than in *rae3*, suggesting that RAE2 also regulates AtALMT1-independent pathway to modulate low Pi response. Furthermore, unlike RAE3 that regulates *STOP1* mRNA export, mutating *RAE2* did not affect *STOP1* mRNA accumulation in the nucleus, although STOP1 protein level was reduced in *rae2*. Introduction of *rae1* mutation into *rae2* mutant background could partially recover the deficient phenotypes of *rae2*. Together, our results demonstrate that RAE2 and RAE3 play overlapping but distinct roles in the modulation of Al resistance and low Pi response.

## Introduction

Aluminum (Al) toxicity represents a major constraint for crop production on acid soils, which comprise more than 30% of the world’s arable land (von Uexkull and Mutert, 1995). To grow on the acid soils, plants have evolved multiple mechanisms to detoxify Al. One key Al-resistance mechanism is that plants can exude organic acids, including malate, citrate, and oxalate, to chelate and detoxify Al (Ma et al., 2001; Ryan et al., 2001; Liu et al., 2014). The model plant *Arabidopsis* (*Arabidopsis thaliana*) secretes both malate and citrate for the Al detoxification, albeit malate plays a more important role in Al resistance than citrate (Hoekenga et al., 2003, 2006; Liu et al., 2009). Genes involved in the exudation of malate and citrate were first identified in crops, which encode anion transporters belonging to the Al-activated malate transporter (ALMT) family and the multidrug and toxic compound extrusion (MATE) family, respectively (Sasaki et al., 2004; Furukawa et al., 2007; Magalhaes et al., 2007). In *Arabidopsis*, *ARABIDOPSIS THALIANA ALUMINUM ACTIVATED MALATE TRANSPORTER 1 (AtALMT1)* and *ARABIDOPSIS THALIANA MULTIDRUG AND TOXIC EXTRUSION (AtMATE)* are mainly responsible for root malate and citrate secretion in response to Al stress, respectively (Hoekenga et al., 2006; Liu et al., 2009).

The zinc-finger transcription factor SENSITIVE TO PROTON RHIZOTOXICITY 1 (STOP1) plays a critical role in Al resistance, which is achieved mainly through the regulation of *AtALMT1* expression (Iuchi et al., 2007). STOP1 also induces the expression of *AtMATE* and *ALUMINUM SENSITIVE 3* (*ALS3*) (Sawaki et al., 2009). ALS3 and ARABIDOPSIS THALIANA SENSITIVE TO ALUMINUM RHIZOTOXICITY (AtSTAR1) form a functional ATP-binding cassette (ABC) transporter to be localized at the tonoplast for the Al detoxification although the underlying mechanism is still unclear (Larsen et al., 2005; Huang et al., 2010; Dong et al., 2017). Recently, *GLUTAMATE DEHYDROGENASE 1 AND 2 (GDH1 and GDH2)*, which regulate Al resistance probably through maintaining cellular pH homeostasis under Al stress conditions, are also demonstrated to be the direct targets of STOP1 (Tokizawa et al., 2021). The transcription of *STOP1* is not responsive to Al stress but its encoded protein can be induced by the Al stress (Iuchi et al., 2007; Zhang et al., 2019). We recently discovered that F-box proteins REGULATION OF ATALMT1 EXPRESSION 1 (RAE1) and RAE1 HOMOLOG 1 (RAH1) can facilitate STOP1 ubiquitination and promote its degradation *via* the ubiquitin-26S proteasome pathway, which is important for balancing Al resistance and plant growth (Zhang et al., 2019; Fang et al., 2021b). STOP1 is also subjected to SUMO modification through the SUMO E3 ligase SIZ1-dependent and SIZI-independent pathways, and is deSUMOylated by the SUMO proteases EARLY IN SHORT DAYS 4 (ESD4) (Fang et al., 2020, 2021a; Xu et al., 2021). The altered levels of STOP1 SUMOylation would affect STOP1 activity and stability, which in turn influences the expression of STOP1-downstream genes and Al resistance (Fang et al., 2020, 2021a; Xu et al., 2021).

Besides Al resistance, STOP1-AtALMT1 pathway is also involved in the regulation of low phosphate (Pi) response (Balzergue et al., 2017; Mora-Macias et al., 2017). Under low Pi conditions, plants can remodel root architecture, such as the inhibition of primary root growth and the increase in lateral root and root hair densities, to enhance Pi uptake. In *stop1* or *Atalmt1*, the low Pi-induced root growth inhibition is impaired (Balzergue et al., 2017; Mora-Macias et al., 2017). AtALMT1-mediated malate release is proposed to promote apoplastic iron (Fe) toxicity and consequently cause the inhibition of root growth when Pi is deficient. In addition, *LOW PHOSPHATE* ROOT 1 AND 2 (*LPR1* and *LPR2*) encoding multi-copper oxidases with ferroxidase activity were demonstrated to play a critical role in low Pi-induced root growth inhibition by the promotion of Fe accumulation and callose deposition in the meristem of the primary root (Svistoonoff et al., 2007; Muller et al., 2015). Brassinosteroid (BR) signaling pathway and CLE14 peptide act upstream and downstream of LPR1/LPR2 to modulate low Pi response, respectively (Singh et al., 2014, 2018; Gutierrez-Alanis et al., 2017). The P5-type ATPase PHOSPHATE DEFICIENCY RESPONSE 2 (PDR2) interacts genetically with LPR1/LPR2 and plays a negative role in low Pi-induced inhibition of root growth (Ticconi et al., 2009).

THO is a conserved multisubunit complex in yeast (*Saccharomyces cerevisiae*), plants, and animals, which is functionally linked to transcription, mRNA processing, and export (Aguilera, 2005; Luna et al., 2012; Heath et al., 2016). Yeast THO complex consists of four strongly interacting subunits of Tho2, Hpr1, Mft1, and Thp2 (Chavez et al., 2000), while plant THO complex is composed of at least six subunits: HPR1/THO1/EMU1, THO2, TEX1/THO3, THO5A/B, THO6, and THO7A/B, which do not contain the orthologs of yeast Mft1 and Thp2 (Furumizu et al., 2010; Jauvion et al., 2010; Yelina et al., 2010). The core THO complex can associate with additional proteins such as the mRNA export factors UAP56 and Aly to form TREX (Heath et al., 2016). Several subunits of the plant THO complex, including HPR1, TEX1/THO3, THO6, and THO2, have been reported to regulate the accumulation of small RNAs including small interfering RNAs (siRNAs) and microRNA (miRNA), which consequently influence the plant morphology, root-associated acid phosphatase activity, female germline specification, and coumarin scopolin accumulation (Furumizu et al., 2010; Jauvion et al., 2010; Yelina et al., 2010; Francisco-Mangilet et al., 2015; Tao et al., 2016; Su et al., 2017; Doll et al., 2018). HPR1 was also documented to regulate disease resistance and senescence probably through modulating mRNA export (Pan et al., 2012). Recently, Guo et al. (2020) found that HPR1 is involved in the regulation of Al resistance partly through the modulation of nucleocytoplasmic *STOP1* mRNA export. It is unclear whether other subunits of the THO complex play a similar role to HPR1 in the regulation of the Al resistance and *STOP1* mRNA export.

To identify the genes involved in the regulation of *AtALMT1* and/or *STOP1*, we previously generated a reporter line containing a fusion gene of *AtALMT1* promoter with luciferase (LUC) reporter gene to screen mutants showing altered LUC signal. Through the screen, we have identified three *regulation of AtALMT1 expression* (*RAE*) genes (Zhang et al., 2019; Fang et al., 2020; Guo et al., 2020), which include *RAE3* that encodes HPR1. In this study, we identified *RAE2* encoding TEX1, another subunit of the THO complex, by using the same screening system. We found that RAE2 plays a similar role to RAE3 in the regulation of STOP1 protein accumulation and the expression of STOP1-downstream genes, but unlike RAE3, RAE2 does not regulate *STOP1* mRNA export. Furthermore, RAE2 modulation of low Pi response involves a different mechanism from RAE3.

## Materials and Methods

### Plant Materials and Growth Conditions

The wild-type (WT) *Arabidopsis* plant (Columbia background; Col-0) contains a homozygous *AtALMT1* promoter-driven *LUC* transgene (*pAtALMT1:LUC*) (Zhang et al., 2019). *rae2* (*tex1*), *rae3* (*hpr1-7*), and *rae1* (*rae1-1*) mutants with altered LUC signal were screened from an ethyl methanesulfonate (EMS)-mutagenized library carrying the *pAtALMT1:LUC* reporter gene previously (Zhang et al., 2019). The T-DNA insertion line *Atalmt1* (SALK_00962) was derived from the Nottingham *Arabidopsis* Stock Centre (NASC).^1^

*Arabidopsis* seeds were surface-sterilized with 6% sodium hypochlorite solution and stratified at 4°C for 3 days in the dark. Seeds were then sown on 1.2% agar medium containing the full-strength Hoagland nutrient and 1% sucrose for 7 days. For luminescence signal detection, 7-day-old seedlings were used for the observation by a CCD imaging apparatus (Lumazone P1300B, Roper Scientific). Plants were grown on nutrient agar plates or one-tenth-strength Hoagland nutrient solution in a growth chamber (CU-36L4, Percival) or grown on soils in a growth room at 22°C with 14 h of light (100 μmol m^–2^ s^–1^; Philips TLD26W865 cool daylight tubes) and 10 h of darkness.

### Measurement of Malate Secretion and Al Content in Roots

Five-day-old seedlings of WT, *rae2*, and *rae3*/*hpr1* grown on 0.4% gellan gum (G1910, Sigma-Aldrich) medium containing the full-strength Hoagland nutrient and 1% sucrose were pretreated with a 2% MGRL nutrient solution containing 1% sucrose at pH 4.8 for 2 h and then exposed to the same solution containing 0 or 20 μM Al at pH 4.8 for 12 h. Root exudates were collected for malate measurement by the NAD/NADH enzymatic cycling method (Hampp et al., 1984). For the determination of Al content, three-week-old seedlings of WT and *rae2* grown on the one-tenth-strength Hoagland solution were pretreated with 0.5 mM CaCl_2_ solution for 6 h at pH 4.8 and then treated with 0.5 mM CaCl_2_ solution containing 0 or 30 μM Al for 12 h at pH 4.8. Al content in roots was determined according to previous methods (Ligaba-Osena et al., 2017; Zhang et al., 2019). The Al concentration in a diluted solution was measured by inductively coupled plasma mass spectrometry (ICP-MS; PerkinElmer NexION300D).

### Evaluation of Al Resistance and Low Pi Response

A soaked gel medium was used to evaluate Al resistance according to a previous method (Larsen et al., 2005) with some modifications. Briefly, a nutrient gel medium consisting of 50 mL (pH 5.0) of 0.25 mM (NH_4_)_2_SO_4_, 1 mM KNO_3_, 0.2 mM KH_2_PO_4_, 2 mM MgSO_4_, 1 mM Ca(NO_3_)_2_, 1 mM CaSO_4_, 1 μM MnSO_4_, 5 μM H_3_BO_3_, 0.05 μM CuSO_4_, 0.2 μM ZnSO_4_, 0.02 μM NaMoO_4_, 0.1 μM CaCl_2_, 0.001 μM CoCl_2_, 1% sucrose, and 0.4% gellan gum (G1910, Sigma-Aldrich) was first prepared and then soaked with 30 mL of the same nutrient solution without gellan gum containing 0, 0.5, 0.75, or 1 mM AlCl_3_ at pH 3.6. After soaking for 2 days, the solution was removed and the gel medium was dried for seed sowing. After 7 days of growth on the gel medium, seedlings were imaged and root lengths were measured by ImageJ software. Relative root growth expressed as root length with Al treatment/root length without Al × 100 was used to assess Al resistance.

To evaluate the response to low Pi, seeds were sown on 0.4% gellan gum medium (pH 5.7) containing the full-strength Hoagland nutrient and 1% sucrose with 40 μM Fe(III)-EDTA and 0, 20, or 1,000 μM NH_4_H_2_PO_4_. After growth for 7 days, the seedlings were photographed and the relative root growth was used for evaluating the sensitivity to low Pi.

### Cloning of *RAE2*

The *rae2* mutant was crossed with WT to produce an F2 population for genetic analysis and mapping-by-sequencing. High-throughput DNA sequencing was performed by using the Illumina HisSeq4000 system that produces 150-bp paired-end reads, which were carried out by a commercial company (Shanghai Hanyu Biotech) (accession number in NCBI: SRR14277220). The MutMap method (Abe et al., 2012) was used to identify the candidate region of *rae2* with slight modifications. The detailed procedure for data processing and map of candidate mutant gene had been described previously (Zhang et al., 2019). To confirm the candidate region of *rae2*, derived cleaved amplified polymorphic sequence (dCAPS) markers were developed and used for the linkage analysis of F2 plants with decreased LUC signal (Supplementary Table 1).

For the complementation test of *rae2*, a DNA fragment harboring a 2-kb promoter and gene sequence of *TEX1* (At5g56130) was amplified (Supplementary Table 2) and cloned into the pCAMBIA3301 vector. The construct was transformed into *rae2* mutant through *Agrobacterium tumefaciens* (strain GV3101)-mediated transformation method. Single-locus homozygous transgenic lines were used for the complementation test.

### RNA Isolation and Expression Analysis

To compare the expression of Al-resistance genes among WT and various mutants, seedlings grown on 1.2% agar medium as described earlier for 9 days were pretreated with a 0.5 mM CaCl_2_ solution at pH 4.8 for 6 h and then treated with the same solution containing 0 or 30 μM AlCl_3_ at pH 4.8 for 12 h. Roots were excised for RNA extraction and expression analysis. To determine *RAE3* expression in various tissues, roots, old and young rosette leaves, cauline leaves, stems, flowers, and siliques of Col-0 wild-type plants were harvested for RNA extraction, respectively. Total RNA were extracted by the TaKaRa MiniBEST plant RNA Extraction Kit (Cat # 9769). About 1 μg of total RNAs was used for the synthesis of the first-strand cDNAs using the HiScript 1^st^ Strand cDNA Synthesis Kit (Vazyme Biotech) after digestion with DNase I. Diluted cDNA products were subjected to the real-time RT-PCR analysis using the SYBR Green Master Mix (Vazyme Biotech) and gene-specific primers (Supplementary Table 2). The reference gene *UBQ10* was used as an internal control for the expression analysis.

### Subcellular Localization Analysis

The *RAE2*/*TEX1* coding sequence was amplified and fused in frame with *GFP* at the N- or C-terminals of *RAE2* under the control of *35S* promoter in pCAMBIA1300 vector. Each resultant vector was transformed into *Arabidopsis* protoplasts for the subcellular localization analysis. GFP fluorescence in the protoplasts was observed by a confocal laser scanning microscope (Leica SP8).

### Nuclei Isolation and Expression Analysis

Two-week-old plants of WT, *rae2*, and *rae3* grown on a one-tenth-strength Hoagland nutrient solution were pretreated with a 0.5 mM CaCl_2_ solution at pH 4.8 for 6 h and then treated with the same solution containing 0 or 30 μM AlCl_3_ at pH 4.8 for 12 h. Root nuclei of each line were isolated according to a previous method (Folta and Kaufman, 2006) with slight modifications. The detailed process for the root nuclei isolation has been described previously (Guo et al., 2020). The isolated nuclei were subjected to RNA extraction and expression analysis. The SAND family gene *AT2G28390* was used as an internal control.

### GUS Activity Assay

To create *pRAE2:GUS* transgenic lines, the 2-kb promoter of *RAE2*/*TEX1* was amplified by a primer pair (Supplementary Table 2) and inserted into pORE-R2 vector harboring the GUS reporter gene. The construct was then introduced into Col-0 plants by *Agrobacterium*-mediated transformation method. A single-locus homozygous transgenic line was used for GUS activity assay. The transgenic seedlings grown on a one-tenth-strength Hoagland nutrient solution for around 3 weeks were stained with a commercialized GUS staining solution (161031; O’Biolab Co., Ltd., Beijing, China) for overnight at 37°C. To investigate whether GUS activity was affected by Al stress, seven-day-old transgenic seedlings were pretreated with a 0.5 mM CaCl_2_ solution at pH 4.8 for 6 h and subsequently treated with the same solution (pH 4.8) containing 0 or 30 μM Al for 12 h. The roots were then stained with the GUS staining solution for 2 h at 37°C. Stained tissues were photographed with a stereomicroscope (SZX12, Olympus) equipped with a camera (DP20, Olympus).

### Immunoblot Analysis

Twelve-old-day seedlings of Col-0 WT, *rae2*, and *rae3* harboring *pSTOP1:STOP1-HA* transgene were pretreated with a 0.5mM CaCl_2_ solution at pH 4.8 for 6 h and then treated with the same solution containing 0 or 30 μM Al at pH 4.8 for 12 h. Total proteins were extracted from the roots or shoots of each line using a extraction buffer composed of 20 mM Tris–HCl (pH 7.4), 300 mM NaCl, 5 mM MgCl_2_ (pH 8.0), 5 mM DTT, 0.5% NP-40, 50 μM MG132 (A2585; APExBIO, United States), and 1× Complete Protease inhibitor tablets EDTA-free (5892791001; Roche). Immunoblot analysis was performed to compare STOP1-HA protein level using anti-HA antibody (H3663; Sigma-Aldrich).

## Results

### Identification of *rae2* Mutant Showing a Reduced Expression of STOP1-Regulated Genes

**FIGURE 1:**
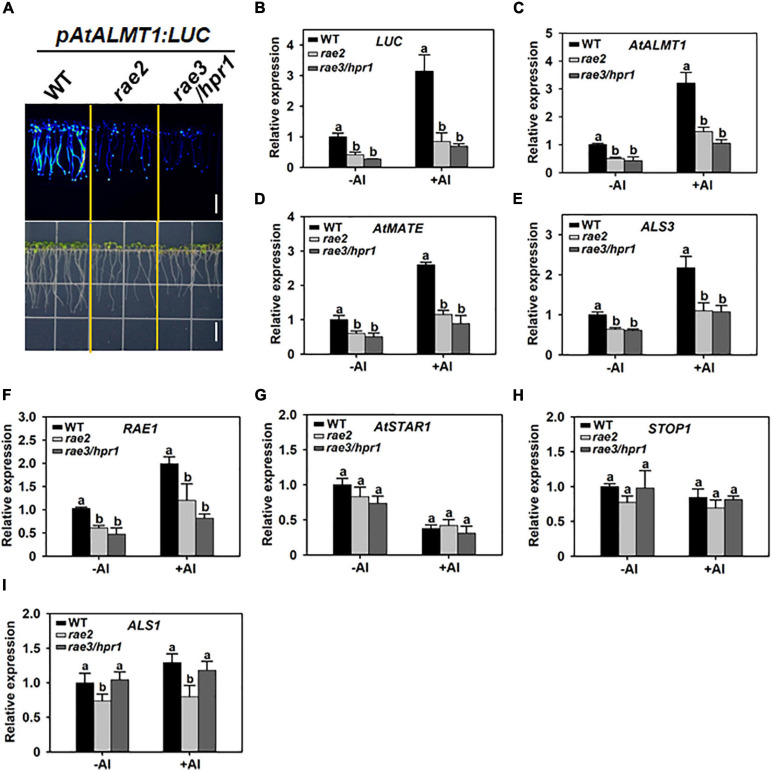
Mutation of *RAE2* decreases the expression of STOP1-regulated genes. **(A)** Decreased LUC signal of *pAtALMT1:LUC* in *rae2* mutant compared with wild type (WT). Bar = 7 mm. **(B–F)** Reduced mRNA expression of STOP1-regulated genes in *rae2* and *rae3*/*hpr1*, which include *LUC* reporter gene **(B)**, *AtALMT1* **(C)**, *AtMATE* **(D)**, *ALS3* **(E)**, and *RAE1* **(F)**. **(G–I)** Comparison of the expression levels of non-STOP1-target genes in WT, *rae2*, and *rae3*, which include *AtSTAR1* **(G)**, *STOP1* **(H),** and *ALS1* **(I)**. Seven-day-old seedlings were exposed to 0 (–Al) or 30 μM Al (+Al) for 12 h, and then the roots were excised for the expression analysis. Data shown are means ± SD of three biological replicates. Different letters at each treatment indicate the values significantly different (*P* < 0.05, ANOVA followed by Tukey’s test).

We previously conducted a forward genetic screen on an EMS-mutagenized population, which harbors the *AtALMT1* promoter-driven luciferase reporter gene (*pAtALMT1:LUC*), and identified a series of *regulation of AtALMT1 expression* (*rae*) mutants with altered LUC signal (Zhang et al., 2019). In this study, we characterized one of these mutants, called *rae2*, which displayed a decreased LUC signal similar to previously reported *rae3*/*hpr1* mutant (Figure 1A). The expression levels of the *LUC* transgene and the endogenous *AtALMT1* gene were then compared in the roots of the wild type (WT), *rae2*, and *rae3* under different Al conditions. Compared with WT, *rae2* showed a reduced expression of *LUC* and *AtALMT1* at a similar level to *rae3* under both 0 (–Al) or 30 μM Al (+Al) conditions (Figures 1B,C).

To examine whether the *rae2* mutation influences the expression of other Al-resistance genes, we compared the expression levels of *AtMATE*, *ALS3*, *RAE1*, *AtSTAR1*, *STOP1*, and *ALS1* in WT, *rae2*, and *rae3*. Like *rae3*, *rae2* showed a decreased expression of the three STOP1-regulated genes *AtMATE*, *ALS3*, and *RAE1* (Figures 1D–F), while the expression of *AtSTAR1* and *STOP1*, which are not regulated by STOP1, was not affected in *rae2* and *rae3* (Figures 1G,H). The expression of *ALS1* not targeted by STOP1 was slightly reduced in *rae2* but not in *rae3* (Figure 1I). Together, these results demonstrate that RAE2 is involved in the regulation of the expression of STOP1-downstream genes.

### Mutation of *RAE2* Diminishes Al Resistance and Low Phosphate Response

**FIGURE 2:**
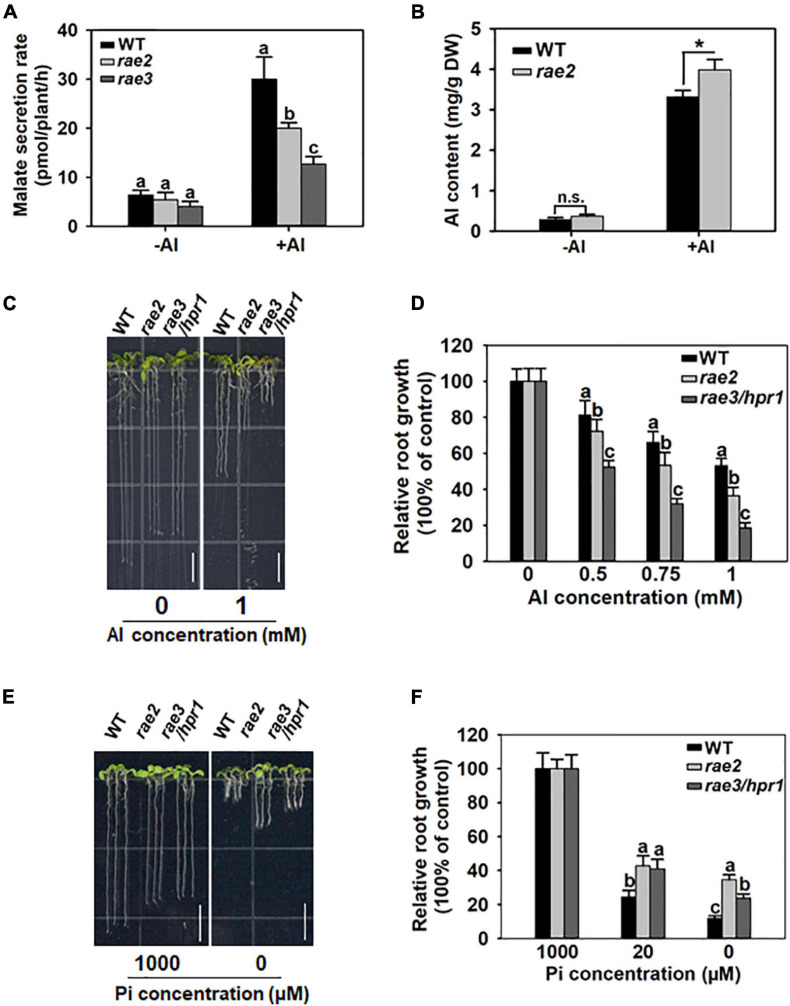
Mutation of *RAE2* reduces Al resistance and low Pi response. **(A)** Malate secretion was reduced in *rae2* compared to WT under +Al conditions. Five-day-old seedlings of WT, *rae2*, and *rae3* were treated with 0 (–Al) or 20 μM Al (+Al) for 12 h, and then root exudates were collected for malate content determination. Values are means ± SD of three biological replicates. **(B)** *rae2* mutant accumulated a higher level of Al than WT. Three-week-old plants of WT and *rae2* were exposed to 0 (–Al) or 30 μM Al (+Al) at pH 4.8 for 12 h, and the roots were harvested for the measurement of Al content. Values are means ± SD of three biological replicates. Asterisks indicate that the values are statistically different (Student’s *t*-test, *P < 0.001). **(C,D)** Representative images **(C)** and relative root growth **(D)** of WT, *rae2*, and *rae3* at different Al concentrations. Seedlings of WT, *rae2*, and *rae3* were grown on a soaked gel medium containing 0, 0.5, 0.75, or 1 mM Al for 7 days, and then the relative root growth was calculated to determine their Al resistance. Values are means ± SD (*n* = 16–20). Bar = 7 mm. **(E,F)** Images **(E)** and quantitative data **(F)** of low Pi-response phenotype in WT, *rae2*, and *rae3*. Seedlings were grown on gellan gum medium containing 0, 20 or 1,000 μM Pi for 7 days, and the relative root growth was used to evaluate the low Pi response. Bar = 7 mm. Values are means ± SD (*n* = 15–20). Different letters at each treatment indicate that the values are significantly different (*P* < 0.05, ANOVA followed by Tukey’s test).

Because the *rae2* mutation reduced *AtALMT1* expression, we compared the level of malate secretion in WT, *rae2*, and *rae3*. Without Al treatment, the amount of malate secreted in *rae2* decreased slightly but not significantly from that in WT (Figure 2A), although the expression of *AtALMT1* was significantly decreased in *rae2* compared to WT (Figure 1C), which could be attributed to the fact that Al stress is required for the trigger of AtALMT1-mediated malate secretion (Hoekenga et al., 2006; Iuchi et al., 2007). In the presence of Al, the level of Al-activated malate secretion in *rae2* was lower than that in WT, but higher than that in *rae3* (Figure 2A). In accordance with the decreased exudation of malate in *rae2*, the mutant accumulated a higher level of Al than the WT under Al treatment conditions (Figure 2B).

We then compared Al resistance phenotype in WT, *rae2*, and *rae3*. In the absence of Al, the root length of *rae2* and *rae3* was similarly shorter than that of WT (Figure 2C). Nevertheless, under Al stress conditions, the root growth of *rae2* was inhibited more than that of WT (Figures 2C,D), indicating that *rae2* was more sensitive to Al toxicity than WT. Compared to *rae3*/*hpr1*, the Al-sensitive phenotype in *rae2* was weaker (Figures 2C,D).

AtALMT1-mediated malate secretion has been reported to play a positive role in low phosphate (Pi)-induced root growth inhibition (Balzergue et al., 2017; Mora-Macias et al., 2017). To investigate whether the *rae2* mutation affected low Pi response, we grew the seedlings of WT, *rae2*, and *rae3* under different Pi levels. The result showed that *rae2* was less sensitive to low Pi-induced inhibition of root growth than WT (Figures 2E,F), consistent with the reduced secretion of malate in *rae2* compared to WT (Figure 2A). Nevertheless, although the amount of malate exudation in *rae2* was more than that in *rae3*, *rae2* was less sensitive to low Pi than *rae3* (Figures 2E,F), suggesting that the *rae2* mutation also influences the malate secretion-independent pathway to modulate low Pi response.

### *RAE2* Encodes TEX1, a Core Subunit of the THO/TREX Complex

A genetic analysis of *rae2* was conducted by using an F2 population from a cross between WT and *rae2* mutant. Observation of LUC signal in 213 F2 plants showed that 52 plants displayed a reduced LUC signal, while the remaining 161 plants had normal LUC signal. The ratio of the number of plants with reduced LUC signal to the number with normal LUC signal fits to 1:3 (χ^*2*^ = 0.013, *P* = 0.91), implying that the reduced LUC expression in *rae2* was controlled by a single recessive gene.

**FIGURE 3:**
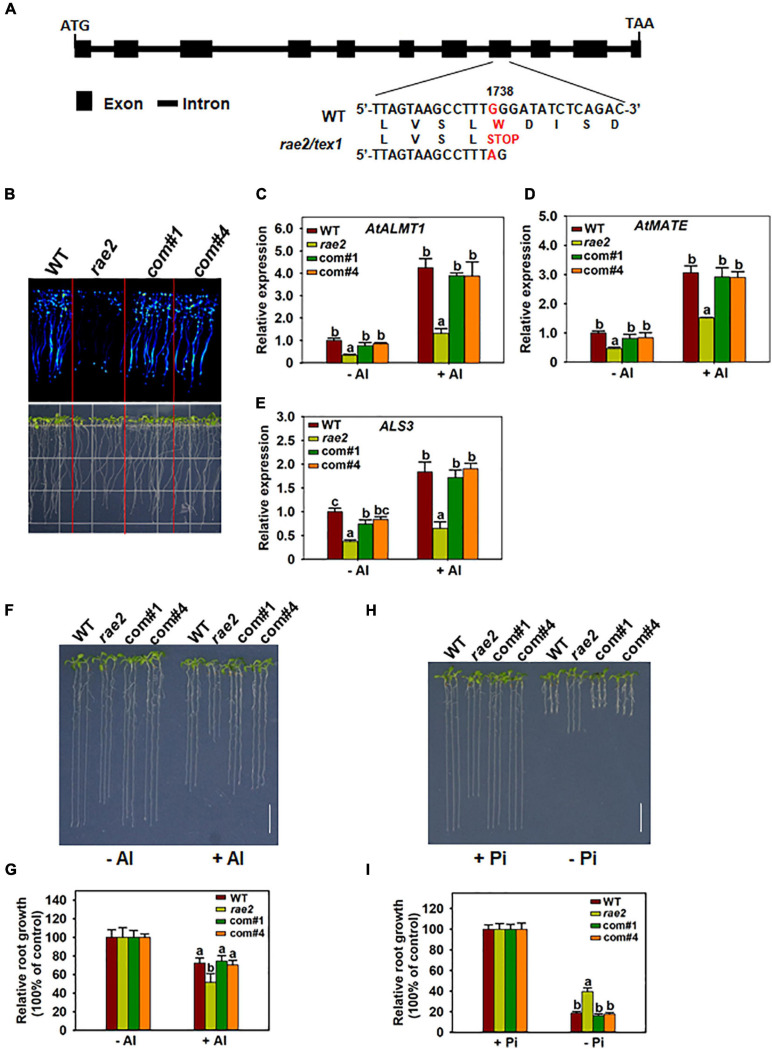
Complementation test of *rae2* mutant. **(A)** Gene structure and mutation sites of *RAE2*. Boxes and horizontal lines between the boxes indicate the coding regions and introns, respectively. A single-nucleotide substitution from G to A was occurred at +1738 from the start codon in *rae2*, which causes an amino acid change from tryptophan to stop codon. **(B–E)** The decreased LUC signal **(B)** and expression of STOP-regulated genes in *rae2*, including *AtALMT1* **(C)**, *AtMATE* **(D)**, and *ALS3* **(E)**, were rescued in two complementation lines. Roots of 7-day-old seedlings were exposed to 0 (–Al) or 30 μM Al (+Al) for 12 h for the expression analysis. Values are means ± SD (*n* = 3). **(F,G)** Complementation of Al resistance phenotype. Representative images **(F)** and relative root growth **(G)** of WT, *rae2*, and complementation lines at different Al concentrations. Values are means ± SD (*n* = 18–23) **(H,I)** Complementation of low Pi response phenotype. Representative images **(H)** and relative root growth **(I)** of WT, *rae2*, and complementation lines at different Pi concentrations. Values are means ± SD (*n* = 19–22). Means with different letters are significantly different (*P* < 0.05, ANOVA followed by Tukey’s test).

To clone the *RAE2* gene, pooled DNA from the 52 F2 plants with reduced LUC signal as described earlier was subjected to the whole-genome sequencing. We used the WT DNA as a control, which was sequenced previously (Zhang et al., 2019). We mapped *rae2* to a small region of chromosome 5 (Supplementary Figure 1) through MutMap method (Abe et al., 2012). We then developed two derived cleaved amplified polymorphic sequence (dCAPS) markers within the candidate region of *rae2* on the basis of the mutations occurred in *rae2* (Supplementary Table 1) to perform a linkage analysis in 36 F2 plants with the reduced LUC signal. The T02 marker displayed a linkage to the LUC signal phenotype with two recombinants, while the T01 marker, developed on a G-to-A substitution at +1,738 bp from the start codon of At5g56130 (*TEX1*), was completely linked to the mutant phenotype (Figure 3A, Supplementary Table 1). This substitution caused an amino acid change from tryptophan to stop codon in *TEX1* in *rae2* (Figure 3A).

To further confirm that *RAE2* is *TEX1*, we conducted a complementation test on *rae2* by transforming a WT *RAE2* with 2-kb promoter and the gene sequence into the mutant. The reduced LUC signal in *rae2* was fully rescued in two independent complementation lines (Figure 3B). The decreased expression of STOP1-regulated genes, including *AtALMT1*, *AtMATE*, and *ALS3*, in the mutant was also recovered in the two lines (Figures 3C–E). We then compared Al resistance and low Pi response in WT, *rae2*, and the two complementation lines. The result showed that the reduced Al resistance and low Pi response in *rae2* were fully rescued in the two complementation lines (Figures 3F–I). Together, these results indicate that mutation of *TEX1* is responsible for the defective phenotypes in *rae2*.

Since RAE2 and RAE3 encode different subunits of the THO complex and are both involved in the regulation of Al resistance and low Pi response, we attempted to generate *rae2 rae3* double mutant by making a cross between the two single mutants *rae2* and *rae3*. Genotyping 52 progenies of *rae2*/*RAE2 rae3*/*rae3* plants showed that 16 progenies had *RAE2*/*RAE2 rae3*/*rae3* genotype, while the remaining 36 progenies possessed *rae2*/*RAE2 rae3*/*rae3* genotype. We were unable to identify *rae2 rae3* double mutant plants, suggesting that mutations of both *RAE2* and *RAE3* would cause embryonic lethality.

### Expression Pattern and Subcellular Localization of RAE2

**FIGURE 4:**
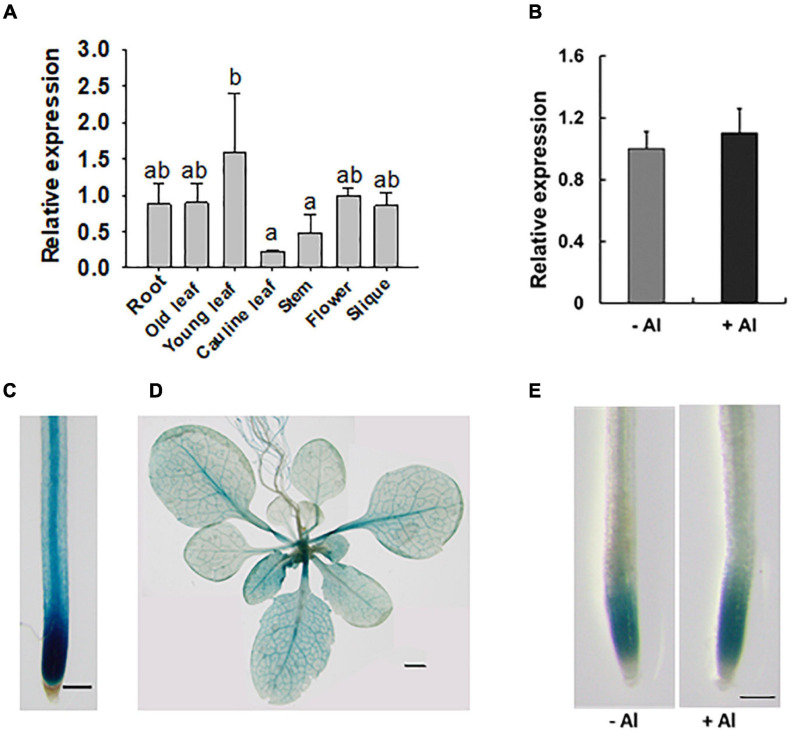
Expression pattern of *RAE2.* **(A)** Expression analysis of *RAE2* in roots, old and young leaves, cauline leaves, stems, flowers, and siliques. Values are means ± SD (*n* = 3). Means with different letters are significantly different (*P* < 0.05, ANOVA followed by Tukey’s test). **(B)** Effect of Al stress on *RAE2* expression. Seven-day-old seedlings were exposed to 0 (–Al) or 30 μM Al (+Al) for 12 h, and the roots were sampled for the expression analysis. Values are means ± SD (*n* = 3). **(C–E)** Detection of GUS activity in *pRAE2:GUS* transgenic lines. **(C)** GUS expression is preferentially detected in the root tips and central vascular tissues of mature root zones. Scale bar = 200 μm. **(D)** GUS expression in the shoots of three-week-old plants. Scale bar = 1 mm. **(E)** GUS expression in roots of 7-day-old seedlings in response to 0 (–Al) or 30 μM Al (+Al) for 12 h. Scale bar = 200 μm.

*RAE2* was well expressed in all the tissues examined (Figure 4A), with relatively lower expression in cauline leaves and stems compared to other tissues. The expression of *RAE2* was not affected by Al stress (Figure 4B). To analyze the expression pattern of RAE2 in detail, we fused *RAE2* promoter with *GUS* reporter gene and then introduced the construct into WT background. GUS staining in a homozygous transgenic line showed that GUS signal was preferentially detected in the tip regions of the roots (Figure 4C). In the mature root zones and leaves, GUS activity was mainly detected in the vascular tissues (Figures 4C,D). Consistent with mRNA expression data of *RAE2*, the level of GUS signal was not responsive to Al stress (Figure 4E).

To determine the subcellular localization of RAE2, we made constructs of *35S:RAE2-GFP* and *35S:GFP*-*RAE2* and then transformed each construct into *Arabidopsis* protoplasts. Both RAE2-GFP and GFP-RAE2 green fluorescent signals were predominantly detected in the nucleus, whereas the signal of GFP control was found in both the nucleus and the cytoplasm (Supplementary Figure 2). These results indicate that RAE2 is localized in the nucleus, which is in agreement with a previous report (Sorensen et al., 2017).

### The *rae2* Mutation Reduces STOP1 Protein Accumulation Not Through Modulating *STOP1* mRNA Export

**FIGURE 5:**
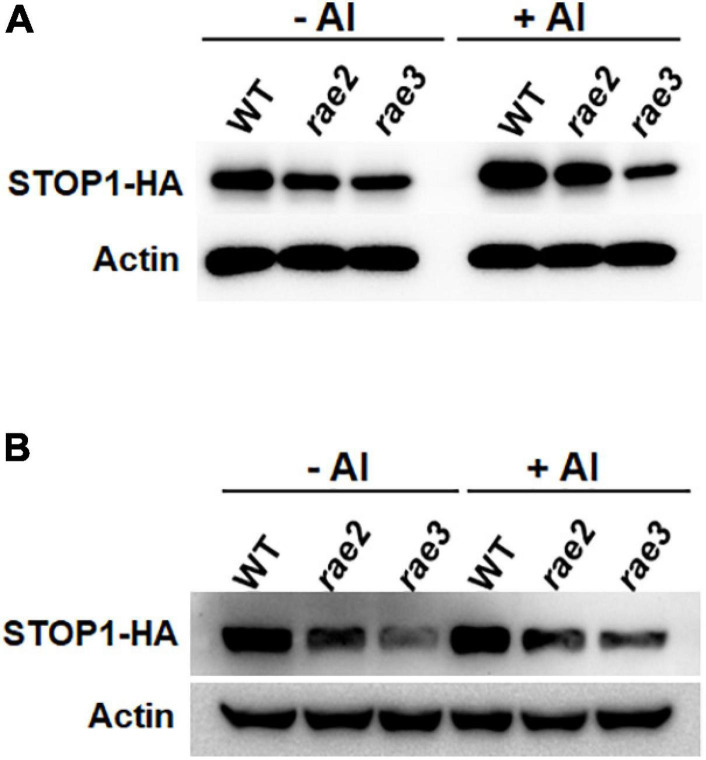
Mutation of *RAE2* reduces STOP1 protein accumulation. **(A,B)** Comparison of STOP1-HA protein level in roots **(A)** and shoots **(B)** of WT, *rae2*, and *rae3* harboring *pSTOP1:STOP1-HA* transgene. Seedlings with 12 days old were exposed to 0 (–Al) or 30 μM Al (+Al) for 12 h. The STOP1-HA and internal control actin was detected by anti-HA and anti-actin antibodies, respectively.

Since the expression of STOP1-regulated genes was decreased in *rae2* (Figure 1), we examined whether the *rae2* mutation influenced STOP1 protein accumulation. We crossed a previously generated transgenic line of *pSTOP1:STOP1-HA* (Zhang et al., 2019) with *rae2* to introduce the transgene into the mutant background. We compared the STOP1-HA protein level in Col-0 WT, *rae2*, and *rae3*. The result showed that STOP1-HA protein level in the roots was lower in *rae2* than in WT under both –Al and +Al conditions (Figure 5A). The reduced STOP1-HA protein accumulation in *rae3* was more prominent than that in *rae2*. We also determined the STOP1-HA protein level in the shoots, and the result showed that the STOP1-HA protein accumulation was reduced in the shoots as well (Figure 5B).

**FIGURE 6:**
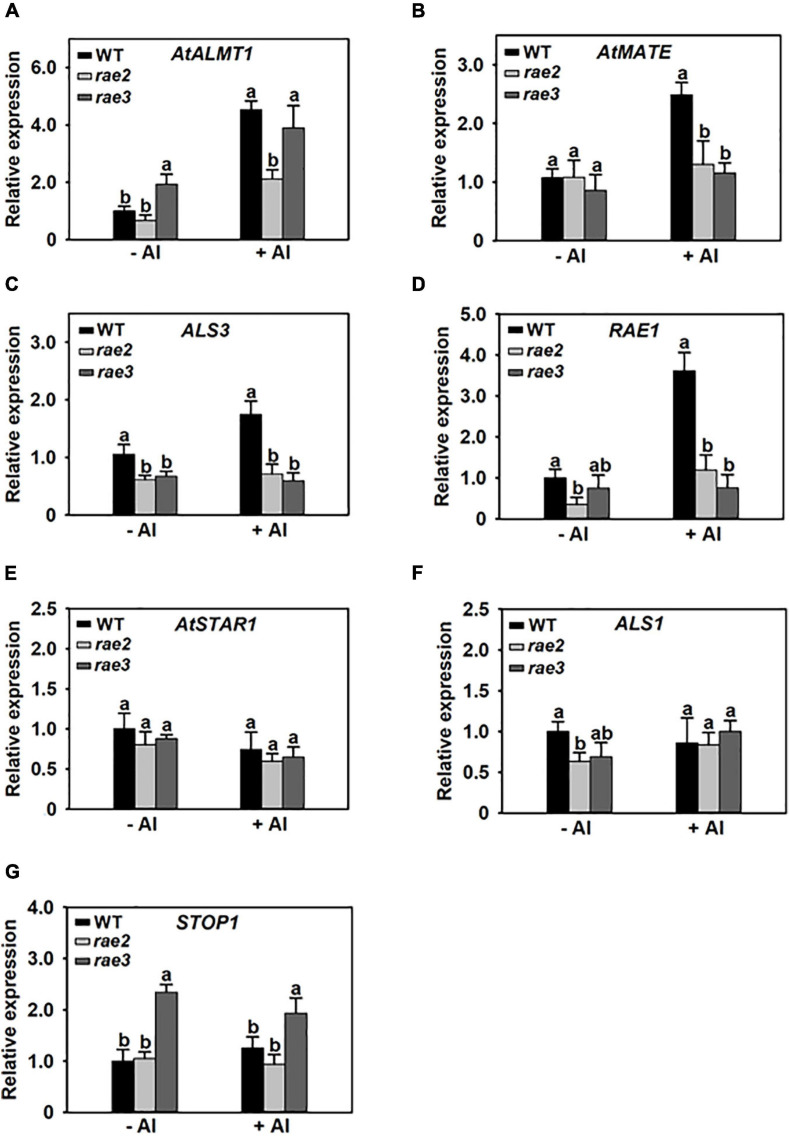
The *rae2* mutation does not influence *STOP1* mRNA export. Three-week-old plants of WT, *rae2*, and *rae3* were treated with 0 (–Al) or 30 μM Al (+Al) for 12 h, and then the root nuclei were isolated for the real-time RT-PCR analysis of Al-resistance genes, which include *AtALMT1* **(A)**, *AtMATE* **(B)**, *ALS3* **(C)**, *RAE1* **(D)**, *AtSTAR1* **(E)**, *ALS1* **(F)**, and *STOP1* **(G)**. Values are means ± SD (*n* = 3). Means with different letters are significantly different (*P* < 0.05, ANOVA followed by Tukey’s test).

To investigate whether RAE2 modulates STOP1 protein accumulation *via* a similar mechanism to RAE3/HPR1, which regulates nucleocytoplasmic *STOP1* mRNA export (Guo et al., 2020), we isolated the nuclei of WT, *rae2*, and *rae3*, and then quantified the expression levels of the Al-resistance genes in the nucleus. Generally, the levels of nuclear mRNA of STOP1-regulated genes were similarly decreased in *rae2* and *rae3* under Al stress conditions, except that the expression of *AtALMT1* in the nucleus was reduced in *rae2* but not in *rae3* compared to WT (Figures 6A–D). The nuclear mRNA levels of *AtSTAR1* and *ALS1* not targeted by STOP1 were similar among the three lines (Figures 6E,F). Noticeably, although *rae3* accumulated more *STOP1* mRNA in the nucleus than WT as previously reported (Guo et al., 2020), the level of nuclear *STOP1* mRNA in *rae2* did not differ from that in WT (Figure 6G). Together, these results demonstrate that RAE2 is involved in the modulation of STOP1 protein accumulation, which might be not through regulating nucleocytoplasmic *STOP1* mRNA export.

### Introduction of *rae1* Mutation Partially Rescues the Defective Phenotypes of *rae2*

**FIGURE 7:**
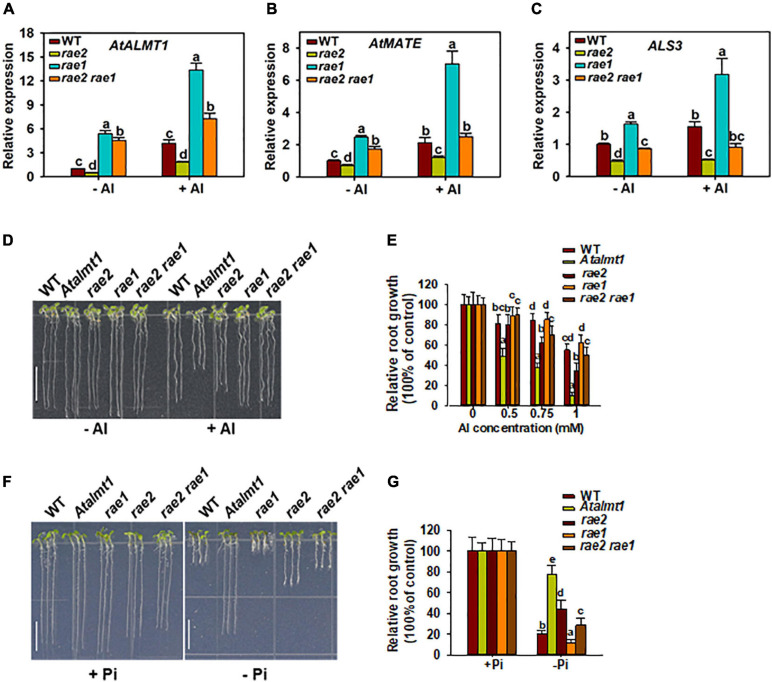
Introduction of *rae1* mutation partially restores the defective phenotypes of *rae2.* **(A–C)** Expression analysis of STOP1-regulated genes, including *AtALMT1* **(A)**, *AtMATE* **(B)** and *ALS3* **(C)**, in WT, *rae2*, *rae1*, and *rae2 rae1*. Seedlings with 7 days old were exposed to 0 (–Al) or 30 μM Al (+Al) for 12 h, and then roots were excised for the expression analysis. Values are means ± SD (*n* = 3). **(D,E)** Representative images **(D)** and Al resistance **(E)** of WT, *Atalmt1*, *rae2*, *rae1*, and *rae2 rae1* under different Al concentrations. Values are means ± SD (*n* = 13–18). **(F,G)** Representative images **(F)** and relative root growth **(G)** of WT, *Atalmt1*, *rae2*, *rae1*, and *rae2 rae1* at 20 (–Pi) or 1,000 Pi (+Pi). Scale bar = 7 mm. Values are means ± SD (*n* = 39–56). Means with different letters are significantly different (*P* < 0.05, ANOVA followed by Tukey’s test).

To investigate whether the decreased expression of STOP1-regulated genes is responsible for the reduced Al resistance and low Pi response in *rae2*, we introduced *rae1* mutation into the *rae2* mutant background *via* crossing. *RAE1* encodes an F-box protein that negatively regulates the expression of STOP1-downstream genes through the modulation of STOP1 stability (Zhang et al., 2019). Expression analysis showed that the decreased expression of STOP1-regulated Al resistance genes, *AtALMT1* and *AtMATE*, in *rae2* was fully recovered in *rae2 rae1* double mutant compared to WT, while that of *ALS3* was partially recovered (Figures 7A–C). We then compared the Al resistance and low Pi response in WT, *Atalmt1*, *rae2*, *rae1*, and *rae2 rae1*. The results showed that the *rae1* mutation could partially rescue the reduced Al resistance in *rae2* (Figures 7D,E). The reduced response to Pi deficiency in *rae2* was also partially rescued by the introduction of *rae1* mutation (Figures 7F,G).

## Discussion

The THO complex is conserved across a wide range of organisms. Components of THO have been reported to regulate mRNA export or the trafficking of small RNA precursors to be involved in diverse biological processes in plants (Furumizu et al., 2010; Jauvion et al., 2010; Yelina et al., 2010; Francisco-Mangilet et al., 2015; Tao et al., 2016; Doll et al., 2018). We previously discovered that the THO complex component RAE3/HPR1 regulates *STOP1* mRNA export to be involved in the regulation of Al resistance (Guo et al., 2020). In this study, we found that another component of the THO complex, RAE2/TEX1, is also involved in regulating Al resistance. We showed that the expression of STOP1-downstream genes and STOP1 protein accumulation were reduced in *rae2* and the introduction of *rae1* mutation into the *rae2* background could partially rescue the increased sensitivity of *rae2* to Al stress, which suggests that RAE2 modulates Al resistance partially through the regulation of STOP1 protein level. Nevertheless, unlike RAE3/HPR1, RAE2 does not regulate *STOP1* mRNA export (Figure 6). The underlying mechanism as to how RAE2 regulates STOP1 protein accumulation is still unknown. THO components are also reported to be able to physically associate with 5′ cap-binding complex (CBC), exon junction complex (EJC), and 3′-end processing factors, implying that THO/TREX might be involved in the regulation of 5′-capping, splicing, and 3′-end processing (Cheng et al., 2006; Johnson et al., 2009; Gromadzka et al., 2016; Heath et al., 2016). Whether RAE2 regulates STOP1 accumulation through the modulation of *STOP1* mRNA processing remains to be investigated in the future.

Compared to *rae3*, the increased Al sensitivity in *rae2* is less prominent (Figures 2C,D). Although *rae2* and *rae3* showed a decreased expression of *AtALMT1* at a similar level (Figure 1C), the reduction in malate secretion was stronger in *rae3* than in *rae2* (Figure 2A), which could be attributed to the fact that RAE3 instead of RAE2 is also involved in the regulation of *AtALMT1* mRNA export (Figure 6A; Guo et al., 2020). These results suggest that the different Al sensitivity between *rae3* and *rae2* may be due to their difference in the level of malate exudation. In contrast, although AtALMT1-mediated malate exudation plays a positive role in low Pi-triggered root growth inhibition (Balzergue et al., 2017; Mora-Macias et al., 2017), the root growth was less inhibited in *rae2* than in *rae3* under low Pi conditions (Figures 2E,F), suggesting that RAE2 also regulates other genes to modulate low Pi response. In addition to AtALMT1, LPR1/LPR2 and PDR2 have also been demonstrated to play critical roles in the regulation of low Pi response by modulating Fe accumulation in root tips (Svistoonoff et al., 2007; Ticconi et al., 2009; Muller et al., 2015). CLE14 peptide and BR signaling pathway components BKI1 and BES1/BZR1 were found to be involved in the low Pi response as well (Singh et al., 2014, 2018; Hirayama et al., 2018). It remains to be determined whether RAE2 is involved in the regulation of these genes required for low Pi response.

Knockout of *THO2* encoding a subunit of THO complex causes embryonic or early seedling lethality (Furumizu et al., 2010; Jauvion et al., 2010; Francisco-Mangilet et al., 2015), indicating that THO2 is essential. By contrast, homozygous *rae2* and *rae3* mutants are viable, but the *rae2 rae3* double mutant could not be obtained, suggesting that RAE2 and RAE3 play redundant roles in the regulation of plant growth and that plant THO/TREX complex is likely to be a dynamic structure in which RAE2 and RAE3 are interchangeable. Our results demonstrate that RAE2 and RAE3 play overlapping but distinct roles in the regulation of Al resistance and low Pi response. Further work is required to determine whether other subunits of the THO complex are also involved in the modulation of the response to Al and low Pi stresses.

## Data Availability Statement

The original contributions presented in the study are publicly available. This data can be found here: NCBI repository, accession number: SRR14277220.

## Author Contributions

All authors conceived the project. C-FH drafted the manuscript. Y-FZ, JG, and YZ performed the experiments. Y-FZ and JG helped to analyze the data and wrote the manuscript. All authors read and approved the final manuscript.

## Conflict of Interest

The authors declare that the research was conducted in the absence of any commercial or financial relationships that could be construed as a potential conflict of interest.

## Footnotes

1 http://szlapncs01.nottingham.ac.uk/
