# Supplementary material for: The THO/TREX Complex Component RAE2/TEX1 Is Involved in the Regulation of Aluminum Resistance and Low Phosphate Response in Arabidopsis
Source: Front Plant Sci. 2021 Jul 12;12:698443. doi: 10.3389/fpls.2021.698443 (PMC8311497; doi:10.3389/fpls.2021.698443)
Supplement: Supplementary file 1 [file Data_Sheet_1.PDF]

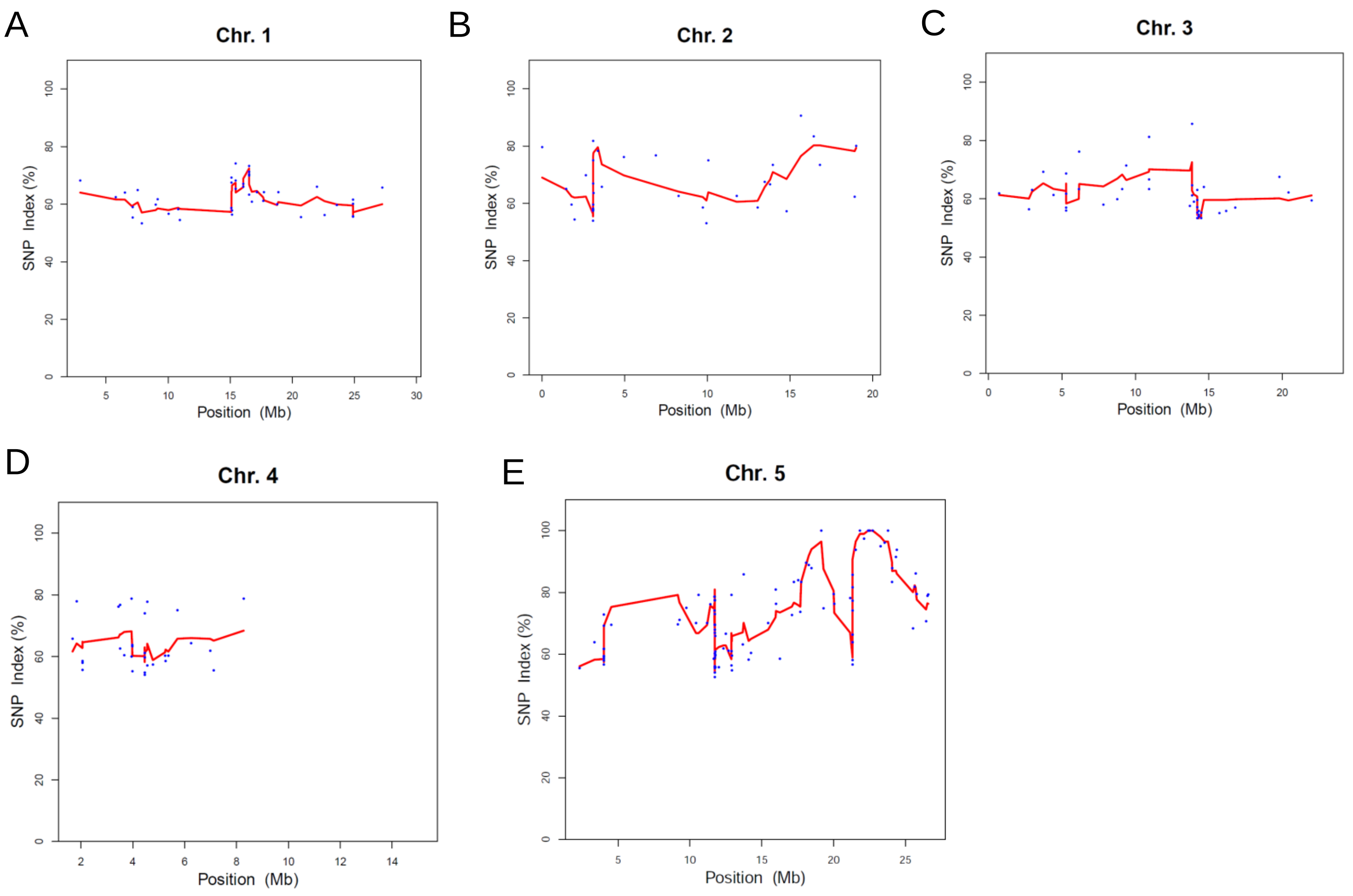

Supplementary Figure 1 | Mutmap analysis of whole-genome sequencing data of pooled F2 mutants on each of five chromosomes. (A) Chromosome 1; (B) Chromosome 2; (C) Chromosome 3; (D) Chromosome 4; (E) Chromosome 5.

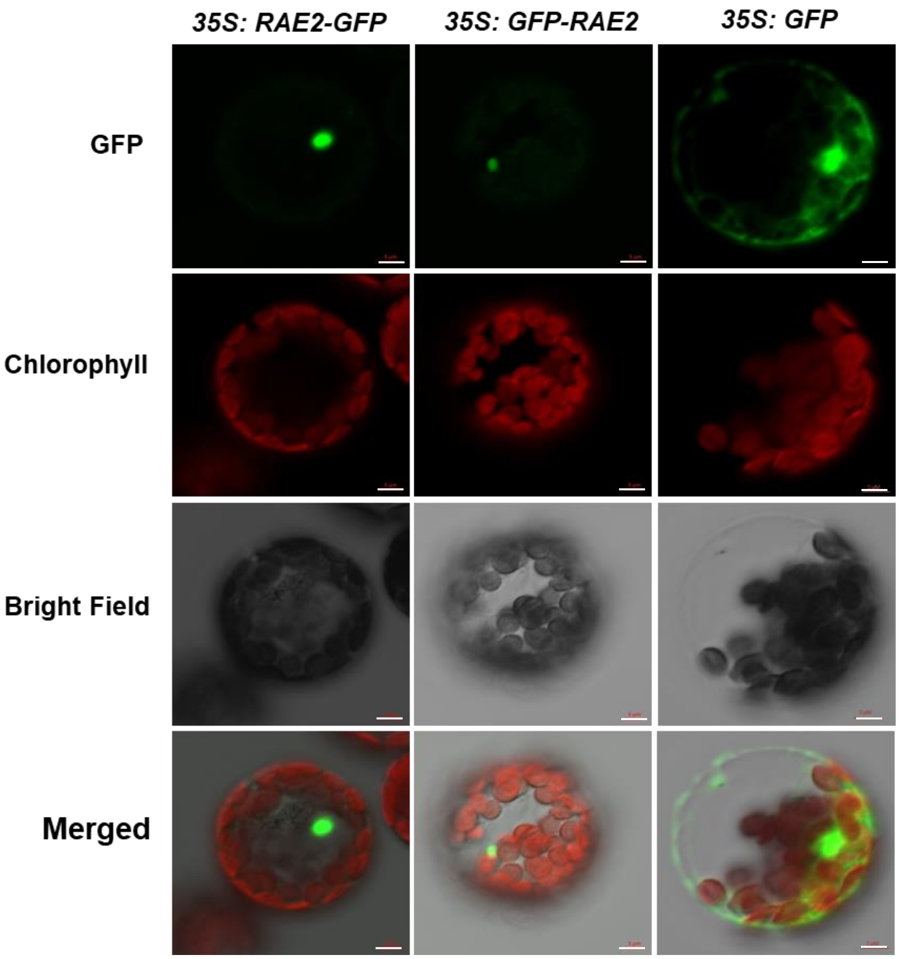

Supplementary Figure 2 | Subcellular localization of RAE2/TEX1. Constructs of *35S:RAE2-GFP* and *35S: GFP-RAE2* weretransformed into *Arabidopsis* protoplasts, respectively, and GFP fluorescence was observed for the determination of subcellular localization by using a confocal laser scanning microscopy. *35S:GFP* was also transformed into the protoplasts and used as a control. Bar=5 μm.

Supplementary Table 1 List of mutation sites in *rae2* mutant and linkage analysis

dCAPS marker	No. of recombinants*	Gene ID	Codon change	
			WT	*rae2*
**T01**	**0**	**AT5G56130**	**TGG**	**TAG**
T02	2	AT5G58900	GCC	ACC

* A total of 36 F2 mutants were used.

Supplementary Table 2 Primers used in this study

Primer name	Forward (5'-3')	Reverse (5'-3')	Purpose
T01	GCTTATTGTTCTGACTGGCCACC	TCGGGAGTGCGGATTCCCCAGTAAGCCTTT	dCAPS marker (XcmI cuts WT)
T02	TGTGGATCAGCTATGTTGGGA	CAACAGCTACATGTGTCCCGC	dCAPS marker (XcmI cuts WT)
LUC	GAAGATGGAACCGCTGGAGA	TGTCCACCTCGATATGTGCA	Real-time RT-PCR
AtALMT1	TGCAAGCTGCGTTGTCGAC	CAAAATCTTGAAGGAAGTGGGAG	Real-time RT-PCR
AtMATE	GTAGCTGGCCAGGCAATACTAGC	GCCACAAACGGAAGTCCTATGC	Real-time RT-PCR
ALS3	CGTATCTCTTCATGGTCTCTGTCG	GTAACTCCGGTGACGGTCATG	Real-time RT-PCR
STOP1	TCACATAGCTCTGTTCCAGGGA	ATCAGTCATTCCAGGCTGTGT	Real-time RT-PCR
AtSTAR1	TTCAAGGGACTGTTGCGGATA	AAGAGCACTTGTTGGTTCATCG	Real-time RT-PCR
ALS1	GCCTCACAGTTGGTTCATCGG	GTCGTTTTTCCTCCACCGCT	Real-time RT-PCR
RAE1	CGAGATTGATGATGAAGGACTGAA	CACTGACCTATAGAGATCAAGTTCACG	Real-time RT-PCR
RAE2/TEX1	AAAGCTTGCTTCGGGTTCTG	CATCCCAAAGACGAACGCTT	Real-time RT-PCR
UBQ10	CGTCTTCGTGGTGGTTTCTAA	GGATTATACAAGGCCCCAAAA	Real-time RT-PCR
SAND	AACTCTATGCAGCATTTGATCCACT	TGATTGCATATCTTTATCGCCATC	Real-time RT-PCR
RAE2-Com	GGGGACAAGTTTGTACAAAAAAGCAGGCTtccaacttcttgccctgg aact	GGGGACCACTTTGTACAAGAAAGCTGGGT CCGAGCTCTCAAAACCA AATATCC	*rae2* complementation
pTEX1-GUS	TGGGGCCCAACGTTCTCGAGACAGAACCGGACATTGGAGA	CGGCCGCAAAGTCGACGAATTCAACAGCTACATGTGTCCCGT	Vector construct of pRAE2:GUS
35S-TEX1-GFP	TCGAGCTCAAGCTATGGAGGAGACGACGATTCCTT	ATCCGGTGGATCCCTACGAGCTCTCAAAACCAAATATCC	Vector construct of 35S:RAE2-GFP
35S-GFP-TEX1	GAGGACACTCGAGATGGAGGAGACACGATTCCTT	CTGCAGTATCGATCGAGCTCTCAAAACCAAATATCC	Vector construct of 35S:GFP-RAE2
